# Magnetic Resonance Imaging Signs of Idiopathic Intracranial Hypertension

**DOI:** 10.1001/jamanetworkopen.2024.20138

**Published:** 2024-07-03

**Authors:** Dagmar Beier, Johanne Juhl Korsbæk, Gabriel Bsteh, Stefan Macher, Wolfgang Marik, Berthold Pemp, Hsiangkuo Yuan, Areeba Nisar, Lisbeth Høgedal, Laleh Dehghani Molander, Snorre Malm Hagen, Christoph Patrick Beier, Simon Bang Kristensen, Rigmor Højland Jensen

**Affiliations:** 1 Department of Neurology, Odense University Hospital, Odense, Denmark; 2 Department of Clinical Research, University of Southern Denmark, Odense, Denmark; 3 OPEN, Odense Patient Data Explorative Network, Odense University Hospital, Odense, Denmark; 4 Danish Headache Center, Department of Neurology, Rigshospitalet-Glostrup, University of Copenhagen, Copenhagen, Denmark; 5 Department of Neurology, Medical University of Vienna, Vienna, Austria; 6 Comprehensive Center for Clinical Neurosciences and Mental Health, Medical University of Vienna, Vienna, Austria; 7 Department of Neuroradiology, Medical University of Vienna, Vienna, Austria; 8 Department of Ophthalmology, Medical University of Vienna, Vienna, Austria; 9 Jefferson Headache Center, Department of Neurology, Thomas Jefferson University, Philadelphia, Pennsylvania; 10 Department of Radiology, Odense University Hospital, Odense, Denmark; 11 Department of Ophthalmology, Odense University Hospital, Odense, Denmark; 12 Department of Ophthalmology, Rigshospitalet, University of Copenhagen, Copenhagen, Denmark; 13 Department of Public Health–Biostatistics, Aarhus University, Aarhus, Denmark

## Abstract

**Importance:**

The magnetic resonance imaging (MRI) criteria currently used to diagnose idiopathic intracranial hypertension (IIH) are based on expert opinion and have limited accuracy. Additional neuroimaging signs have been proposed and used with contradictory results; thus, prospective evidence is needed to improve diagnostic accuracy.

**Objective:**

To provide evidence-based, accurate MRI signs for IIH diagnosis.

**Design, Setting, and Participants:**

This prospective cohort study was conducted from January 2018 to May 2021 with 3 validation cohorts at 2 Danish headache centers and with 3 independent international cohorts. Consecutive patients with suspected IIH were enrolled. Eligibility required the clinical suspicion of IIH, age 18 years or older, and written informed consent. The validation cohorts comprised patients with confirmed IIH from Austria and the US, and patients without IIH from the US. Data analysis was performed from December 2021 to August 2023.

**Exposure:**

Standardized diagnostic workup was performed to classify cases according to current criteria, and blinded evaluation of cerebral radiological diagnostics and papilledema was performed.

**Main Outcomes and Measures:**

The primary outcomes were MRI signs associated with IIH as assessed by univariate analyses. An MRI score estimating papilledema was calculated using machine learning. Internal validation of associations with lumbar puncture opening pressure and outcome and external validation of accuracy were performed in 3 cohorts.

**Results:**

Of 192 eligible patients (185 women [96.4%]; median [IQR] age, 28.0 [23.0-35.0] years), 110 were classified as having IIH, 4 as having probable IIH, and 1 as having suspected IIH without papilledema; 77 did not have IIH and served as controls, with corresponding age, sex, and weight. Papilledema at diagnosis was associated with perioptic subarachnoid space distension (56 patients [68.3%] vs 21 patients [41.2%]), posterior globe flattening (53 patients [66.3%] vs 10 patients [21.3%]), optic nerve disc protrusion (35 patients [30.4%] vs 2 patients [2.3%]), and transverse sinus venous stenosis (75 patients [79.8%] vs 29 patients [46.8%]). The papilledema-estimating MRI score showed optimal balance between sensitivity (49%) and specificity (87%) when 2 of the 3 latter signs were present and was associated with the lumbar puncture opening pressure and ophthalmological outcome. The score showed strong diagnostic accuracy in the external validation cohorts (587 patients; area under the receiver operating characteristic curve, 0.86) and outperformed the current (2013) diagnostic MRI criteria.

**Conclusions and Relevance:**

The findings of this cohort study of patients with IIH and controls suggest that an evidence-based MRI score including posterior globe flattening, optic nerve disc protrusion, and transverse sinus stenosis can estimate the presence of papilledema more accurately than the current diagnostic criteria.

## Key Points

**Question:**

Are there evidence-based magnetic resonance imaging (MRI) signs that improve the accuracy of idiopathic intracranial hypertension (IIH) diagnosis?

**Findings:**

In this cohort study of 192 patients, the presence of 2 of 3 IIH-specific MRI signs (posterior bulb flattening, optic nerve disc protrusion, and transverse sinus venous stenosis) enabled the diagnosis of papilledema in patients with suspected IIH with greater accuracy than with the use of current diagnostic MRI criteria. This diagnostic ability was confirmed in 3 independent cohorts.

**Meaning:**

These findings suggest that 3 IIH-specific MRI signs can be used to diagnose papilledema more accurately than the current diagnostic MRI criteria.

## Teaser

This cohort study seeks to provide accurate magnetic resonance imaging–based criteria for the diagnosis of idiopathic intracranial hypertension.

## Introduction

Idiopathic intracranial hypertension (IIH) is characterized by elevated intracranial pressure of unknown cause, resulting in typical symptoms such as headache and impairment of the optic nerve due to papilledema. It is rare in the general population but disproportionately affects women of childbearing age with obesity (20 cases per 100 000 population).^1,2,3^ Currently, the diagnosis of IIH is based on expert opinion, as summarized in the 2013 diagnostic criteria,^4^ and requires (1) normal neurological examination findings except abducens nerve palsy, (2) normal magnetic resonance imaging (MRI) brain parenchymal and cerebral venography findings, (3) normal cerebrospinal fluid (CSF) composition, (4) elevated lumbar puncture opening pressure (≥25 cm H_2_O in adults), and (5) papilledema. However, when papilledema is absent but conditions 1 to 4 are fulfilled, patients are classified as having IIH without papilledema when presenting with abducens nerve palsy or as having suspected IIH without papilledema when presenting with 3 of 4 MRI criteria (empty sella, flattening of the posterior aspect of the globe, distension of the perioptic subarachnoid space with or without a tortuous optic nerve, and transverse venous sinus stenosis^4^) instead. However, the diagnostic impact of these MRI criteria is vague, because they are neither evidence based nor further specified, resulting in variable interpretation and application; furthermore, they are often difficult to assess with standard MRI examination and require specific thin-slice orbital or pituitary gland sequences. Because of these pitfalls, up to 40% of patients examined outside tertiary centers receive an incorrect diagnosis.^5,6,7,8^ An evidence-based update of the 2013 diagnostic criteria, in which IIH is defined by the presence of 2 of 3 findings (papilledema, opening pressure ≥25 cm H_2_O, and ≥3 neuroimaging signs), has been suggested recently.^9^ With this update, abducens nerve palsy was removed from the criteria, and empty sella was replaced by suprasellar CSF herniation into more than one-third of the sella turcica.^9^ In addition, an increasingly large panel of neuroimaging signs associated with IIH has been proposed, although its use has yielded contradictory results, and these signs have not been validated in a prospective, clinical practice patient cohort.^10,11,12,13,14^ Here, we blindly evaluated a comprehensive panel of neuroimaging signs for IIH diagnosis in a well-defined, meticulously characterized population of patients with clinically suspected IIH and validated our results internally in parts of our patient cohort, as well as externally in patient cohorts from other university hospitals.

## Methods

### Patients and Ethical Considerations

#### Danish Prospective Cohort

This prospective cohort study included patients aged 18 years or older with clinically suspected IIH who were evaluated at 2 tertiary headache centers in Denmark (Odense University Hospital, since January 2018, and the Danish Headache Center, Rigshospitalet-Glostrup, since November 2018).^15^ Clinical IIH suspicion was raised by a senior neurologist or ophthalmologist based on objective findings (papilledema or abducens nerve palsy), clinical symptoms (headache, pulsatile tinnitus, or visual disturbances), and phenotype (young female patient with obesity). Patients with clinically suspected IIH relapse were included only if they presented with active disease (papilledema relapse). The exclusion criteria were secondary pseudotumor cerebri syndrome, pseudopapilledema or optic disc swelling due to other diseases, and incompleteness of the data needed to confirm or exclude the diagnosis. The standardized diagnostic workup included a structured interview; neurological and neuro-ophthalmological examinations; lumbar puncture (with the measurement of the opening pressure and determination of CSF composition); cerebral MRI, magnetic resonance venography, or computed tomography venography examination; routine blood collection; and electrocardiography.^15^ Standardized lumbar punctures were performed as described elsewhere.^9^

According to the 2013 diagnostic criteria,^4^ cases were classified as IIH, probable IIH, IIH without papilledema, suspected IIH without papilledema, and non-IIH (control). Secondary pseudotumor cerebri syndrome was diagnosed in patients fulfilling the diagnostic criteria for IIH but presenting with an independent disease that was likely causing intracranial pressure elevation. Parts of this cohort have been described elsewhere.^9,16,17^ The study was approved by the Ethics Committee of Southern Denmark and the Danish Data Protection Agency. Data are reported in line with the Strengthening the Reporting of Observational Studies in Epidemiology (STROBE) reporting guidelines. All patients gave written informed consent. Additional details are given in the eAppendix in Supplement 1.

#### Independent International Validation Cohorts

Cohort 1 consisted of outpatients requiring brain MRI examinations for reasons other than IIH.^13^ Cohort 2 was a prospective cohort of patients with IIH from the University Hospital Vienna (Vienna, Austria).^18^ Cohort 3 consisted of highly selected patients with difficult-to-treat conditions referred for sinus transversus stenting at the Jefferson Headache Center, Philadelphia, Pennsylvania; data on this cohort have not been published. More detailed information on the international validation cohorts is provided in the eAppendix in Supplement 1.

### Diagnostic Workup for the Danish Prospective Cohort

#### Radiological Diagnostics

Radiological diagnostics were performed in different radiological departments in the Region of Southern Denmark, Zealand, and the Capital Region of Denmark and complied with the 2013 criteria recommendations.^4^ They were partly supplemented by the following sequences: T1-weighted imaging with or without contrast enhancement (1.5 or 3.0 T), thin-slice fat-suppressed orbital sequences, pituitary gland sequences, and cerebral time-of-flight magnetic resonance venography without contrast enhancement or contrast-enhanced computed tomography venography. To avoid interrater bias, a single senior neuroradiologist (L.H.) blinded to the diagnoses reexamined all cerebral images. The images were evaluated with regard to pathological findings, fulfillment of the MRI diagnostic criteria,^4,9^ and a proposed panel of MRI characteristics compiled from the literature^14^ and the authors (ocular globe length and width, optic nerve disc protrusion, optic nerve canal diameter, optic nerve and sheath calibers, posterior pituitary stalk displacement, arachnoid pitting, presence of small meningoceles, inferior cerebellar tonsil position, and Evan index; for definitions and details, see the eAppendix in Supplement 1).

#### Ophthalmological Assessment

Three specific senior consultants (L.D.M. and 2 other individuals who are not coauthors of this article) with experience in the diagnosis of papilledema and pseudopapilledema in the headache centers’ ophthalmology departments conducted all ophthalmological assessments, as described elsewhere.^9^ All unilateral and bilateral visual field defects, including reduced sensitivity, enlarged blind spots, and optic nerve (retinal nerve-fiber layer) atrophy, were defined as long-term ophthalmological sequelae.

### Statistical Analysis

Data analysis was performed from December 2021 to August 2023. The statistical analysis was performed using SPSS statistical software version 29.0 (IBM). The groups were compared using the χ^2^ and Fisher exact test (for frequencies <5) for categorical variables and the Kruskal-Wallis test for continuous variables. The threshold for statistical significance was 2 sided, and the significance level used for all analyses was *P* < .05. When patient data were insufficient for the evaluation of a neuroimaging sign, the patient was excluded from that analysis.

#### Papilledema and IIH-Estimating MRI Score Development

Detailed information about score calculation is provided in the eAppendix in Supplement 1. To identify crucial MRI signs associated with papilledema, we used multivariate logistic regression with least absolute shrinkage and selection operator regularization for the sparse shrinkage of regression coefficients. The analysis was performed using Stata SE statistical software version 17.0 (StataCorp). The binary outcome was papilledema. The following signs, which were part of the 2013 diagnostic criteria or were significantly associated in the univariate analyses, were used as explanatory variables: (1) unilateral or bilateral flattening of the posterior aspect of the globe, (2) unilateral or bilateral distension of the perioptic subarachnoid space, (3) unilateral or bilateral optic nerve disc protrusion, and (4) unilateral or bilateral transverse sinus stenosis along with the 4 categories of (5) herniation of the suprasellar cistern; thus, there was a total of 8 parameters including the intercept. Model performance was summarized in terms of discrimination as measured by the area under the receiver operating characteristics curve (AUROC) along with the E_max statistic proposed by Harrell et al^19^ and Austin et al.^20^ To address the problem of missing predictors, imputation was performed. Bootstrapping was performed to assess internal validity.^21^

#### Score Validation

The diagnostic accuracy of the 2013 MRI criteria^4^ and the MRI score developed in this study was compared. The presence of unilateral or bilateral papilledema was the binary outcome. The AUROC and Youden index (for cutoff value determination) were calculated. This analysis was performed using SPSS statistical software version 29.0 (IBM).

## Results

### Patient Characteristics

The Danish cohort comprised 243 patients (123 patients at Odense University Hospital and 120 patients at Danish Headache Center, Rigshospitalet-Glostrup). Of 192 eligible patients (185 women [96.4%]; median [IQR] age, 28.0 [23.0-35.0] years) included in the analysis, 110 had IIH, 4 had probable IIH, 1 had suspected IIH without papilledema, and 77 did not have IIH and formed the control group.^4^ eFigure 1 in Supplement 1 provides an overview of the diagnostic workflow. The 115 patients with IIH were younger and weighed less than those without IIH (Table 1). As expected, patients with IIH presented at the time of diagnosis with papilledema (114 patients [99.1%] vs 0 patients [0.0%]) and higher lumbar puncture opening pressure (40.6 vs 26.5 cm H_2_O) vs controls (Table 1).

**Table 1.** Patients’ Demographics and Clinical Characteristics

Characteristic	Patients, No. (%)		*P* value
	With IIH (n = 115)	Without IIH (n = 77)	
Diagnosis according to the revised Friedman criteria^4^			
IIH	110 (95.7)	NA	NA
IIH without papilledema	0	NA	
Probable IIH	4 (3.5)	NA	
Suspected IIH without papilledema	1 (0.9)	NA	
Without IIH	NA	77 (100.0)	NA
Sex			
Female	111 (96.5)	74 (96.1)	>.99^a^
Male	4 (3.5)	3 (3.9)	
Body mass index at diagnosis, mean (IQR)^b^	36.4 (31.4-41.5)	38.7 (31.4-43.6)	.02^c^
Age at diagnosis, median (IQR), y	28.5 (23.0-33.0)	32.4 (22.5-40.0)	.005^c^
Lumbar puncture opening pressure at diagnosis, median (IQR), cm H_2_O	40.6 (33.0-50.0)	26.5 (20.0-33.0)	<.001^c^
Optic disc evaluation			
Papilledema	114 (99.1)	0	<.001^a^
Normal	1 (0.9)	77 (100.0)	
Contraception use^d^	48 (42.9)	40 (52.6)	.19^e^

Abbreviations: IIH, idiopathic intracranial hypertension; NA, not applicable.

^a^ Calculated with Fisher exact test.

^b^ Body mass index is calculated as weight in kilograms divided by height in meters squared.

^c^ Calculated with Kruskal-Wallis test.

^d^ Contraception included oral contraceptives, contraceptive coil, intravaginal rings, and contraceptive implants; we excluded 1 pregnant patient and 3 patients treated with artificial insemination from the analysis.

^e^ Calculated with χ^2^ test.

### MRI Characteristics

To evaluate the associations of MRI characteristics with the presence of at least unilateral papilledema, the patients were divided into those with and without papilledema (eFigure 1 in Supplement 1). On their MRI at diagnosis, more patients with than without papilledema presented with unilateral (53 patients [66.3%] vs 10 patients [21.3%]) and bilateral (43 patients [53.8%] vs 5 patients [10.6%]) posterior globe flattening, unilateral (56 patients [68.3%] vs 21 patients [41.2%]) and bilateral (54 patients [65.9%] vs 17 patients [33.3%]) perioptic subarachnoid space distension, and unilateral (35 patients [30.4%] vs 2 patients [2.3%]) and bilateral (22 patients [19.1%] vs 1 patient [1.1%]) optic nerve disc protrusion (Table 2). The bulb width and length, optic nerve tortuosity, optic nerve canal diameter, optic nerve and optic nerve sheath calibers, and absolute diameter of the perineural subarachnoid space did not differ between these groups (Table 2). Three patients underwent contrast-enhanced MRI as part of extended diagnostic workup, and none showed enhancement of the optic nerve (data not shown).

**Table 2.** MRI Characteristics With Regard to the Optic Nerve and the Bulb

Characteristic (No. of patients available for evaluation)^a^	Patients, No. (%)		*P* value
	With papilledema	With normal optic discs	
Flattening of the posterior aspect of the globe present (n = 127)			
No	27 (33.8)	37 (78.7)	<.001^b^
Yes	53 (66.3)	10 (21.3)	
Bilateral flattening of the posterior aspect of the globe (n = 127)			
No	37 (46.3)	42 (89.4)	<.001^b^
Yes	43 (53.8)	5 (10.6)	
Globe measurements, mean (SD), mm (n = 74)			
Width of the right globe, MR orbita	23.0 (0.9)	23.4 (1.1)	.20^b^
Length of the right globe, MR orbita	22.5 (1.1)	22.8 (1.0)	.50^b^
Width of the left globe, MR orbita	23.3 (0.9)	23.4 (1.0)	.90^b^
Length of the left globe, MR orbita	22.2 (1.3)	22.8 (1.1)	.40^b^
Distension of the perioptic subarachnoid space present (>2 mm) (n = 133)			
No	26 (31.7)	30 (58.8)	.002^b^
Yes	56 (68.3)	21 (41.2)	
Bilateral distension of the perioptic subarachnoid space (>2 mm) (n = 133)			
No	28 (34.1)	34 (66.7)	<.001^b^
Yes	54 (65.9)	17 (33.3)	
Tortuous optic nerve present (n = 165)			
No	71 (72.4)	47 (70.1)	.75^b^
Yes	27 (27.6)	20 (29.9)	
Bilateral tortuous optic nerve (n = 165)			
No	75 (76.5)	58 (86.6)	.11^b^
Yes	23 (23.5)	9 (13.4)	
Optic disc protusion present (n = 71)			
No	18 (15.7)	16 (18.2)	<.001^c^
Yes	35 (30.4)	2 (2.3)	
Bilateral optic disc protrusion (n = 71)			
No	31 (27.0)	17 (19.3)	<.001^c^
Yes	22 (19.1)	1 (1.1)	
Other measurements, mean (SD), mm			
Diameter optic canal right (n = 71)	3.7 (0.6)	3.7 (0.5)	.70^d^
Diameter optic canal left (n = 72)	4.0 (0.6)	3.6 (0.5)	.20^d^
Caliber optic nerve right (n = 50)	2.9 (0.5)	2.8 (0.6)	.60^d^
Caliber optic nerve left (n = 49)	2.9 (0.5)	2.9 (0.6)	.90^d^
Caliber optic nerve sheath right (n = 51)	6.4 (1.0)	5.7 (1.0)	.20^d^
Caliber optic nerve sheath left (n = 49)	6.3 (0.9)	5.8 (1.0)	.50^d^
Perineural subarachnoid space right (n = 38)	1.8 (0.5)	1.7 (0.7)	.80^d^
Perineural subarachnoid space left (n = 37)	1.8 (0.4)	1.7 (0.7)	.80^d^

Abbreviation: MRI, magnetic resonance imaging.

^a^ Because evaluation of some parameters required MRI of the orbita, which is not part of the standard diagnostic workup, the number of patients available for evaluation of the different parameters vary.

^b^ Calculated with χ^2^ test.

^c^ Calculated with Fisher exact test.

^d^ Calculated with Kruskal-Wallis test.

The MRI characteristics of the pituitary gland are shown in eTable 1 in Supplement 1. No significant difference was observed between the groups, regardless of the definitions used and signs studied.

The MRI characteristics of the venous sinuses are shown in Table 3. More patients with than without papilledema presented with unilateral (75 patients [79.8%] vs 29 patients [46.8%]) and bilateral (53 patients [56.4%] vs 22 patients [35.5%]) transverse sinus stenosis. In addition, the index of transverse sinus stenosis was significantly higher in the former group. The frequencies of stenoses of other venous sinuses did not differ between groups (Table 3).

**Table 3.** Magnetic Resonance Imaging Characteristics With Regard to the Venous Sinuses

Characteristic (No. of patients available for evaluation)^a^	Patients, No. (%)		*P* value^b^
	With papilledema	With normal optic discs	
Transverse sinus stenosis right (n = 156)			
No	25 (26.6)	34 (54.8)	.001
Yes	69 (73.4)	27 (43.5)	
Hypoplasia	0	1 (1.6)	
Transverse sinus stenosis left (n = 156)			
No	24 (25.5)	36 (58.1)	<.001
Yes	59 (62.8)	24 (38.7)	
Hypoplasia	11 (11.7)	2 (3.2)	
Transverse sinus stenosis present (n = 156)			
No	19 (20.2)	33 (53.2)	<.001
Yes	75 (79.8)	29 (46.8)	
Bilateral transverse sinus stenosis (n = 156)			
No	41 (43.6)	40 (64.5)	.01
Yes	53 (56.4)	22 (35.5)	
Other sinus stenosis right (n = 157)			
No	93 (96.9)	60 (98.4)	.57
Stenosis sinus sigmoideus	3 (3.1)	1 (1.6)	
Other sinus stenosis than sinus transverus/sigmoideus stenosis	0	0	
Other sinus stenosis left (n = 159)			
No	93 (96.9)	62 (98.4)	.55
Stenosis sinus sigmoideus	3 (3.1)	1 (1.6)	
Other sinus stenosis than sinus transversus/sigmoideus stenosis	0	0	
Stenosis of any kind present (n = 158)			
No	15 (15.6)	31 (50.0)	<.001
Stenosis	81 (84.4)	31 (50.0)	
Graduation of transverse sinus stenosis (n = 109)			
67%-83% stenosis	9 (16.4)	3 (5.6)	.01
50%-66% stenosis	9 (16.4)	6 (11.1)	
34%-50% stenosis	14 (25.5)	7 (13.0)	
17%-33% stenosis	8 (14.5)	4 (7.4)	
0%-16% stenosis	2 (3.6)	3 (5.6)	
No stenosis	13 (23.6)	31 (57.4)	
Index of transverse sinus stenosis score (n = 192)			
0	31 (33.0)	39 (62.0)	.006
1	2 (2.1)	4 (6.5)	
2	2 (2.1)	2 (3.2)	
3	1 (1.1)	1 (1.6)	
4	8 (8.5)	5 (8.1)	
6	8 (8.5)	2 (3.2)	
8	1 (1.1)	1 (1.6)	
9	36 (38.3)	8 (12.9)	
12	4 (4.3)	0	
16	1 (1.1)	0	

^a^ Because evaluation of some parameters required supplemental magnetic resonance venography and/or computed tomography venography, the number of patients available for evaluation of the different parameters vary.

^b^ Calculated with the χ^2^ test.

Data on the other proposed MRI characteristics are provided in eTable 2 in Supplement 1. The presence of arachnoid pits, small meningoceles, inferior cerebellar tonsil positioning, and the Evan index did not differ between the groups.

### MRI Score Development

Detailed information about score calculation is provided in the eAppendix in Supplement 1. To develop an MRI score estimating the presence of papilledema, we chose 5 MRI signs significantly associated with papilledema in the univariate analyses or being part of the 2013 diagnostic criteria^4^: suprasellar cistern herniation, posterior globe flattening, perioptic subarachnoic space distension, optic nerve disc protrusion, and transverse sinus venous stenosis. The analysis was performed on an imputed dataset comprising data from 191 patients whose MRI findings enabled the assessment of at least 1 sign. Following tuning of the least absolute shrinkage and selection operator regularization, the model selected 4 predictors (data not shown) with an AUCROC of 69.3% (after correction for bootstrap optimism), allowing for the calculation of an MRI score (raw MRI score; eFigure 2A in Supplement 1). The Youden index for this score was highest for a cutoff value of 87, corresponding to the presence of at least 2 of the 3 major signs, with a sensitivity of 49% and specificity of 87% in patients, whose MRI scans allowed for assessment of all variables (64 of 192 patients; data not shown). Because suprasellar cistern herniation contributed only marginally, we simplified the score to the 3 major signs: transverse sinus venous stenosis, optic nerve disc protrusion, and posterior globe flattening. The presence of 2 of these 3 signs constituted a positive MRI score indicating papilledema (eFigure 2B in Supplement 1).

### Score Validation

On internal validation, the MRI score was associated with the maximum reported lumbar puncture opening pressure (82 patients) (Figure A) and ophthalmological long-term sequelae (83 patients) (Figure B). The maximum reported lumbar puncture opening pressure correlated with the MRI score (Spearman ρ = 0.267; *P* = .02). The MRI score did not differentiate between IIH and secondary pseudotumor cerebri syndrome (221 patients) (Figure C).

**Figure.**
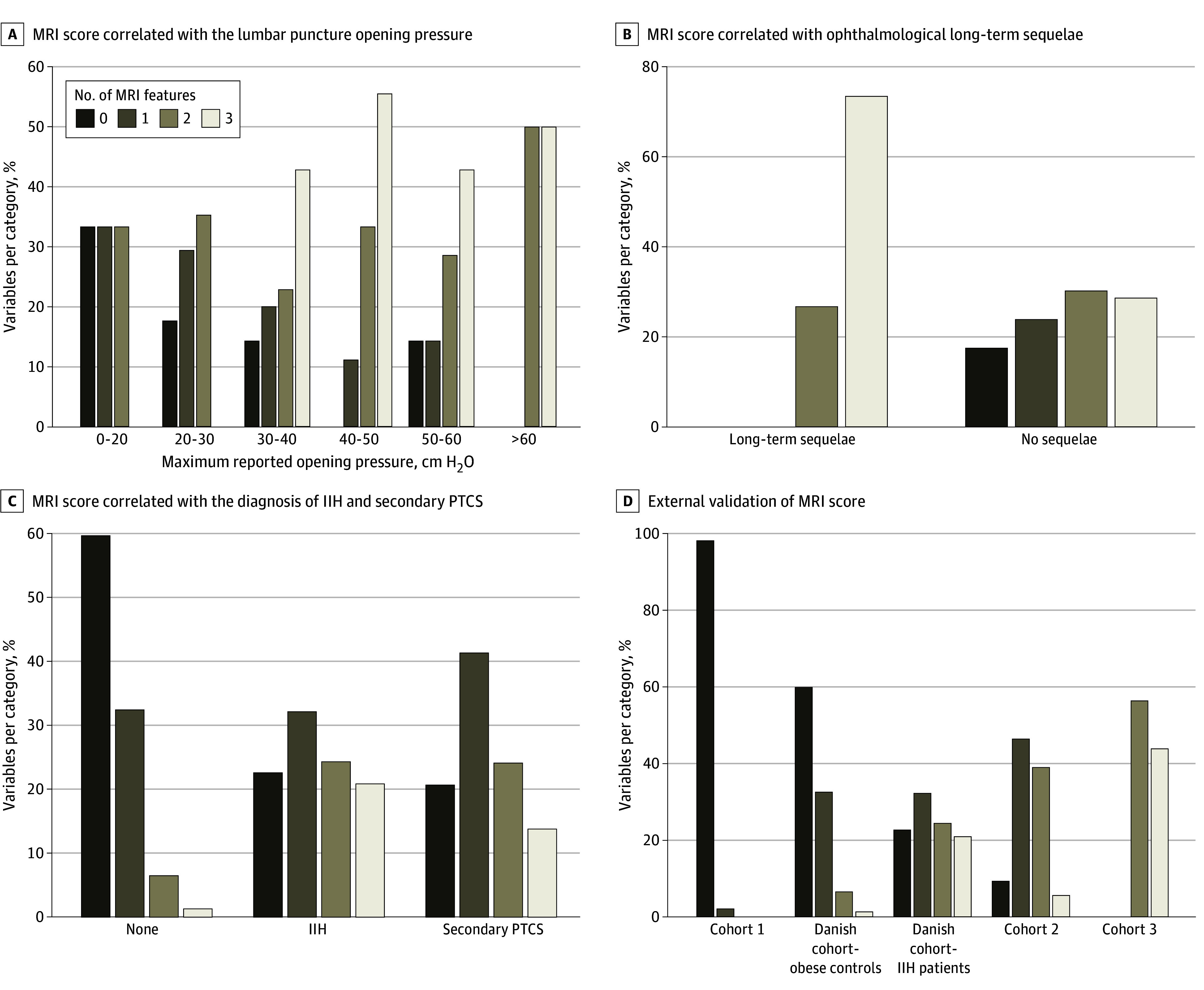
Validation of the Magnetic Resonance Imaging (MRI) Score A and B, MRI scans of patients with clinically suspected idiopathic intracranial hypertension (IIH) were used, allowing for the assessment of all variables without imputation. A, The number of MRI score variables depending on the maximum reported lumbar puncture opening pressure is shown (n = 82; *P* < .001, χ^2^ test for all comparisons). B, The number of MRI score variables depending on the presence of ophthalmological long-term sequelae is shown (n = 83; *P* = .004, χ^2^ test for all comparisons). C, The number of MRI score variables depending on the diagnosis is shown. Because of the small number of patients with secondary pseudotumor cerebri syndrome (PTCS), all patients with clinically suspected IIH were included independent of the quality of their scans and therefore also included those MRI scans that did not allow for sufficient evaluation of all variables (n = 221; *P* < .001, χ^2^ test for all comparisons). D, The number of MRI score variables depending on the diagnosis in 3 independent international patient cohorts is shown (*P* < .001, χ^2^ test for all comparisons).

External validation of the score was difficult, given the lack of published clinically relevant data from similar cohorts. The MRI score was found to correlate strongly with the presence or absence of papilledema in validation cohorts 1 to 3 and in patients with confirmed IIH and controls in the present Danish cohort (Figure D and eTables 3 and 4 in Supplement 1). For the combined cohort comprising all 587 patients and controls, the MRI score had a sensitivity of 47.5% and a specificity of 96.8% (AUROC, 0.86). Positive and negative predictive values were not calculated owing to the heterogeneity of the cohorts and arbitrary prevalence of IIH.

### Score Performance

The ability of the MRI score and 2013 MRI criteria^4^ to estimate papilledema in 2 clinically relevant scenarios was compared using subgroups of the Danish prospective cohort. In the first scenario, data from 78 patients whose MRI scans enabled the assessment of all variables were used. In the second clinical scenario, data from all 203 patients were used, regardless of MRI scan quality. The MRI score outperformed the 2013 MRI criteria in scenarios 1 (AUROC, 0.81 vs 0.67) and 2 (AUROC, 0.76 vs 0.68) (eFigures 3 and 4 in Supplement 1).

## Discussion

We conducted a comprehensive, prospective, cohort study of neuroimaging signs in a clinically relevant cohort of patients with suspected IIH with the aim of obtaining an evidence-based definition of bona-fide neuroradiological signs diagnosing papilledema. In contrast to other studies,^14,22,23,24,25^ this study was prospective and involved evaluation by examiners blinded to radiological and ophthalmological findings. Most important, it included patients with confirmed IIH diagnoses and those with suspected but disproven IIH and corresponding age, weight, and sex, which accurately reflects the clinical practice situation and challenges faced when diagnosing IIH.

Consistent with previous findings, significantly more patients with than without papilledema presented with posterior globe flattening, perioptic subarachnoid space distension, bilateral transverse sinus venous stenoses (index of transverse sinus stenosis score ≥4), and optic nerve disc protrusion.^26,27^ Increased optic nerve sheath diameter has been proposed to be associated with IIH on the basis of a small retrospective study,^28^ and our findings for this variable are strikingly similar to the published data,^23,28^ including our recently reported ultrasound measurements.^29^ In line with others,^23,30^ we found that transverse sinus stenosis was the only sinus venous stenosis associated with papilledema in a clinically relevant manner.

Empty sella, included in the 2013 diagnostic criteria,^4^ did not differ significantly between groups in this study regardless of the definition used. Empty sella is found in healthy individuals^31^ and is associated with obesity^32^ and other diseases^33^; its reported peak incidence is at 30 to 40 years, and it shows female predominance.^33^ Thus, it is likely to appear in young women with obesity and not to be associated directly with IIH. Moreover, few studies describing empty or partially empty sella as a valid neuroimaging sign of IIH were performed prospectively, and many were performed with small or atypical cohorts.^11,12,33^ In some, the modified Dandy diagnostic criteria were used instead of the current diagnostic criteria.^11^ In line with our finding, a recent prospective study^34^ conducted with data from 122 patients with IIH revealed no association between empty sella and the lumbar puncture opening pressure. The present study found no correlation of papilledema with other MRI findings^14,35,36^ proposed to be associated with IIH (eTable 2 in Supplement 1). Our results do not exclude small associations with IIH but do not suggest a high diagnostic yield of these variables.

The MRI score was highly associated with the lumbar puncture opening pressure and both IIH and secondary pseudotumor cerebri syndrome, indicating that its components essentially reflect chronically increased intracranial pressure. These findings, especially the association with the opening pressure, are in line with the highly significant association of the score with long-term ophthalmological outcomes.

The MRI score showed a high degree of specificity for IIH but limited sensitivity in the validation cohorts. Although the low sensitivity constitutes a limitation, the score still outperformed the 2013 diagnostic criteria. Thus, its use would likely reduce the number of asymptomatic patients referred to headache centers because of the detection of single imaging signs, such as (partially) empty sella. It also strongly indicates a risk of developing the full clinical picture of IIH, even in patients without papilledema, justifying ophthalmological follow-up and, when sufficient suspicion of IIH persists, lumbar puncture.

IIH likely represents a continuum of varying degrees of increased intracranial pressure, with and without (ie, before the development of) papilledema. The higher rate of MRI signs and slightly elevated average opening pressure in the non-IIH controls than in the outpatient cohort in this study support this concept implicating IIH and IIH without papilledema being part of the same continuum rather than 2 different entities. In line, IIH without papilledema has been removed in the recently published update of the diagnostic criteria.^9^ Our Danish cohorts included few patients with IIH without papilledema, in contrast with other reports that uniformly report ophthalmological outcomes of IIH without papilledema limiting the impact of this point.^37^

For patients with clinically suspected IIH, lumbar puncture remains an obligatory part of the diagnostic workup to exclude secondary pseudotumor cerebri syndrome, but the MRI score developed in this study may help to reduce the impact of the error-prone measurement of the lumbar puncture opening pressure and the similarity of the numbers of MRI signs in the Danish and Austrian populations, together with the low detection rate in outpatients without papilledema,^13^ suggests that interrater variability is acceptable.

### Limitations

A limitation of the study was the use of different scanners, scanning modalities, and protocols, resulting in relatively small numbers of patients with regard to certain MRI signs. The small numbers themselves, however, were a limitation when using machine learning.

## Conclusions

This study found that the flattening of the posterior aspect of the globe, protrusion of the optic nerve disc, and transverse sinus stenosis constitute an evidence-based MRI score diagnosing papilledema with greater accuracy than the current diagnostic criteria.

## Notes

Supplement 1. **eFigure 1.** Diagnostic work-flow **eFigure 2.** Calculation of the MRI-score **eFigure 3.** Comparison of the MRI-score with the MRI criteria from 2013 **eFigure 4.** Performance of the MRI score **eTable 1.** MRI characteristics with regard to the pituitary gland **eTable 2.** Other MRI characteristics **eTable 3.** Demographics and clinical patient characteristics of the Vienna cohort (cohort 2) **eTable 4.** Demographics and clinical patient characteristics of the USA cohort (cohort 3) **eAppendix.** Supplementary Methods
eReferences

Supplement 2.
Data Sharing Statement
