# Supplementary material for: Magnetic Resonance Imaging Signs of Idiopathic Intracranial Hypertension
Source: JAMA Netw Open. 2024 Jul 3;7(7):e2420138. doi: 10.1001/jamanetworkopen.2024.20138 (PMC11223000; doi:10.1001/jamanetworkopen.2024.20138)
Supplement: Supplement 1. — eFigure 1. Diagnostic work-flow eFigure 2. Calculation of the MRI-score eFigure 3. Comparison of the MRI-score with the MRI criteria from 2013 eFigure 4. Performance of the MRI score eTable 1. MRI characteristics with regard to the pituitary gland eTable 2. Other MRI characteristics eTable 3. Demographics and clinical patient characteristics of the Vienna cohort (cohort 2) eTable 4. Demographics and clinical patient characteristics of the USA cohort (cohort 3) eAppendix. Supplementary Methods eReferences [file jamanetwopen-e2420138-s001.pdf]

Supplemental Online Content

Beier D, Korsbaek JJ, Bsteh G, et al. Magnetic resonance imaging signs of idiopathic intracranial hypertension. *JAMA Netw Open*. 2024;7(7):e2420138. doi:10.1001/jamanetworkopen.2024.20138

eFigure 1. Diagnostic work-flow
eFigure 2. Calculation of the MRI-score
eFigure 3. Comparison of the MRI score with the MRI criteria from 2013
eFigure 4. Performance of the MRI-score
eTable 1. MRI characteristics with regard to the pituitary gland
eTable 2. Other MRI characteristics
eTable 3. Demographics and clinical patient characteristics of the Vienna cohort (cohort 2)
eTable 4. Demographics and clinical patient characteristics of the USA cohort (cohort 3)
eAppendix. Supplementary Methods
eReferences

This supplemental material has been provided by the authors to give readers additional information about their work.

© 2024 Beier D et al. *JAMA Network Open.*

eFigure 1: Diagnostic work-flow

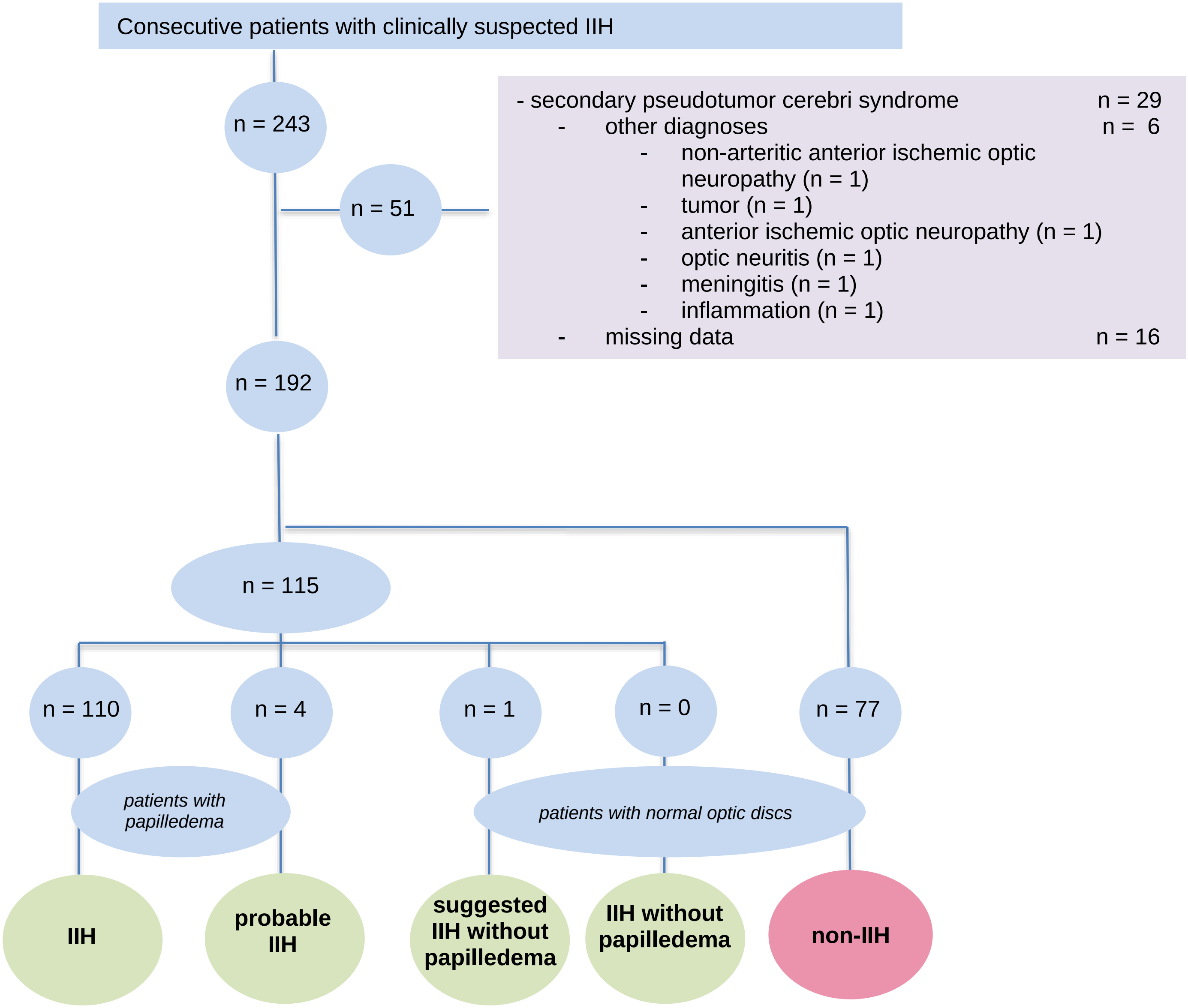

IIH, idiopathic intracranial hypertension

© 2024 Beier D et al. *JAMA Network Open.*

eFigure 2: Calculation of the MRI-score

A Raw MRI-score

➢at least one-sided transverse sinus stenosis / hypoplasia present	+ 47 points
➢at least one-sided optic nerve disc protrusion present	+ 62 points
➢at least one-sided flattening of the posterior aspect of the globe present	+ 65 points
➢herniation of the suprasellar cistern not present	- 1 point
➢herniation of the suprasellar cistern >1/3 present	+ 1 point
Risk of papilledema: 1/(1+exp(-[-0.45+score/100‘))	

➡

B MRI-score with only 3 major MRI signs

➢at least one-sided transverse sinus stenosis / hypoplasia present	+ 1 point
➢at least one-sided optic nerve disc protrusion present	+ 1 point
➢at least one-sided flattening of the posterior aspect of the globe present	+ 1 point
**If ≥ 2 variables are present, papilledema is likely and the MRI-score considered ‘positive‘.**	

© 2024 Beier D et al. *JAMA Network Open.*

eFigure 3: Comparison of the MRI score with the MRI criteria from 2013

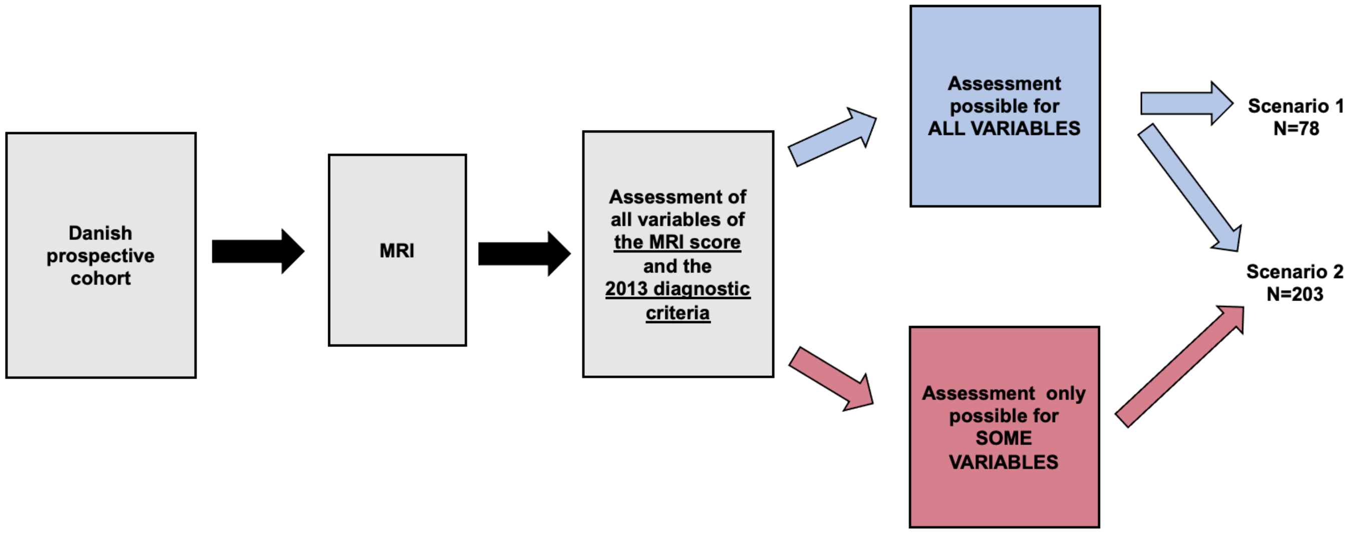

© 2024 Beier D et al. *JAMA Network Open.*

eFigure 4: Performance of the MRI-score

A Scenario 1: Patients whose MRI scan allowed assessment of all variables of both criteria (n=78)

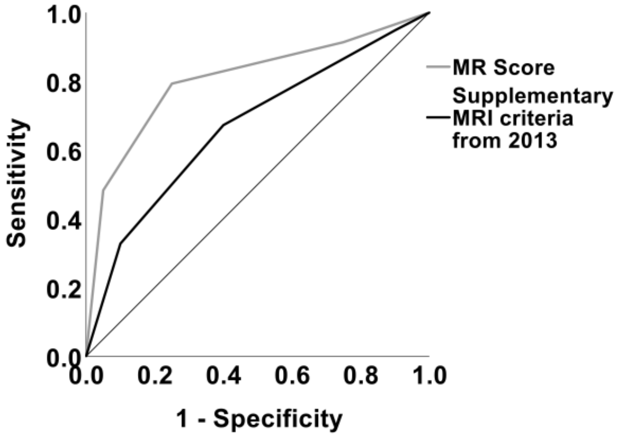

Test Result Variable(s)	Area under the Curve	Significance	AUC 95% Confidence Interval Lower Bound	Upper Bound
Revised Friedman criteria	.669	.025	.537	.801
MR Score	.806	<.001	.701	.910

B Scenario 2: Patients with all kinds of MRI scans not necessarily allowing the assessment of all variables of the respective criteria (n=203)

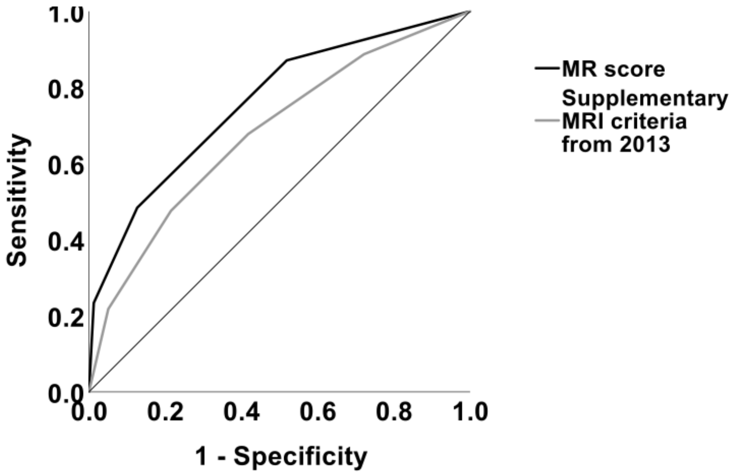

Test Result Variable(s)	Area under the curve	Significance	95% Confidence Interval Lower Bound	Upper Bound
Revised Friedman Criteria	.680	<.001	.606	.754
MR Score	.758	<.001	.692	.824

© 2024 Beier D et al. *JAMA Network Open.*

eTable 1 MRI characteristics with regard to the pituitary gland

		Patients with papilledema	Patients with normal optic discs	P-value	Number of patients available for evaluation
**Empty sella and < 2mm visible pituitary gland tissue, N (%)**	**No**	100 (100.0)	65 (97.0)	P = 0.156#	N = 167
	**yes**	0 (0.0)	2 (3.0)		
**Herniation of the suprasellar cistern corresponding to > 1/3 of the sella height, N (%)**	**no**	33 (33.0)	30 (44.8)	P = 0.124*	N = 167
	**yes**	67 (67.0)	37 (55.2)		
**Herniation of the suprasellar cistern, N (%)**	**no**	23 (23.0)	26 (38.8)	P = 0.151*	N = 167
	**0 - 1/3**	10 (10.0)	4 (6.0)		
	**1/3 - 2/3**	34 (34.0)	17 (25,4)		
	**2/3 - 3/3**	33 (33.0)	20 (29.9)		
**Posterior displacement of the pituitary stalk, N (%)**	**no**	51 (54.3)	42 (65.6)	P = 0.154*	N = 158
	**yes**	43 (45.7)	22 (34.4)		

Note: As evaluation of some parameters required MRI with pituitary sequences, which is not part of the standard diagnostic work-up, the number of patients available for evaluation of the different parameters vary.
**Chi square test; #Fisher's exact test*

© 2024 Beier D et al. *JAMA Network Open.*

eTable 2 Other MRI characteristics

		Patients with papilledema	Patients normal optic discs	P-value	Number of patients available for evaluation
**Arachnoid pits, N (%)**	**no**	79 (81.4)	56 (83.6)	P = 0.724¤	N = 164
	**yes**	18 (18.6)	11 (16.4)		
**Small meningoceles, N (%)**	**no**	99 (100.0)	68 (100.0)	P=1.0*	N = 167
	**yes**	0 (0.0)	0 (0.0)		
**Inferior position of the cerebellar tonsils, mean (standard deviation), mm**		0.375 (1.784)	0.000 (0.0)	P = 0.175#	N= 162
**Evan's index, mean (standard deviation)**		0.248 (0.023)	0.248 (0.024)	P = 0.114#	N = 159

Note: As evaluation of the different parameters was dependent on the quality of the MRI images the number of patients available for evaluation of the different parameters vary,
*¤Chi square test; #Kruskal Wallies test; * Fisher's exact test*

© 2024 Beier D et al. *JAMA Network Open.*

eTable 3 **Demographics and clinical patient characteristics of the Vienna cohort (cohort 2)**

		Patients classified as<br><br>IIH, probable IIH, IIH-WOP, suggested IIHWOP<br><br>N=84
**Diagnosis according to the revised Friedman criteria (ref)**<br><br>**N (%)**	**IIH**	70 (83.3)
	**IIHWOP**	4 (4.8)
	**probable IIH**	5 (6.0)
	**suggested IIHWOP**	5 (6.0)
**N (%)**	**Non-IIH**	0 (0.0)
**Sex N (%)**	**female**	74 (88.1)
	**male**	10 (11.9)
**BMI at diagnosis**<br>**Mean (25 percentile – 75 percentile)**		30.7 (27.3 – 39.0)
**Age at diagnosis**<br>**Mean (years) (25 percentile – 75 percentile)**		33.5 (25.5 – 38.5)
**Lumbar puncture opening pressure (OP) at diagnosis**<br>**Mean (cm H_2O) (25 percentile – 75 percentile)**		31.5 (28.0 – 27.0)
**Papil status**<br>**N (%)**	**papilledema**	75 (89.3)
	**normal**	9 (10.7)

IIH, idiopathic intracranial hypertension; IIHWOP, IIH without papilledema; BMI, body mass index

© 2024 Beier D et al. *JAMA Network Open.*

eTable 4 **Demographics and clinical patient characteristics of the USA cohort (cohort 3)**

		Patients classified as IIH, probable IIH, IIH-WOP, suggested IIHWOP N=16	Patients classified as Non-IIH Patients N=1	Insufficient data N=3
**Diagnosis according to the revised Friedman criteria (ref) N (%)**	**IIH**	10 (62.5)[a]		
	**IIHWOP**			
	**probable IIH**	2 (12.5)		
	**suggested IIHWOP**	3 (18.8)		
**N (%)**	**Non-IIH**		1 (100)	
**N(%)**	**Insufficient data**	1 (6.3)[c]		3 (100)[b]
**Sex N (%)**	**female**	15 (93.7)	1 (100)	3 (100)
	**male**	1 (6.3)		
**BMI at diagnosis Mean (25 percentile – 75 percentile)**		36.1 (27.3 – 56.3)	46.6	30.1 (22.9-37.3)
**Age at diagnosis Mean (years) (25 percentile – 75 percentile)**		37.5 (20.0 – 54.0)	53.0	43.3 (22.0-57.0)
**Lumbar puncture opening pressure (OP) at diagnosis Mean (cm H_2O) (25 percentile – 75 percentile)**		33.6 (22.0 – 60.0)[c]	19.0	29.5 (28.0-31.0)[d]
**Papil status N (%)**	**papilledema**	13 (81.3)		
	**Normal optic disc**	3 (18.7)	1	2[e]

[a] No MRI performed in 1 patient
[b] MRI or/and MRV not performed in 3 patients.
[c] No lumbar puncture performed in 1 patient, which nevertheless fulfilled the diagnostic criteria.
[d] No lumbar puncture performed in 1 patient.
[e] No fundoscopic exam in 1 patient

IIH, idiopathic intracranial hypertension; IIHWOP, IIH without papilledema; BMI, body mass index

© 2024 Beier D et al. *JAMA Network Open.*

eAppendix. Supplementary Methods

Ethical approvals

The study was approved by the Ethics Committee of Southern Denmark (VEK S-20170058) and the Danish Data Protection Agency (nos. 16/20512, 06.06.2016, and 07.04.2017). Data are reported in line with the Strengthening the Reporting of Observational Studies in Epidemiology recommendations[1]. All patients gave written informed consent.

Data availability

Individual, de-identified participant data can be shared with qualified researchers who provide a methodologically sound proposal. It is a legal requirement that a data processing agreement is signed and approved by the data protection office in the Region of Southern Denmark. Raw imaging data are shared locally for technical and legal reasons. Data are available 2 years after publication. Proposals should be directed to the corresponding author.

Radiological diagnostics:

All images were evaluated with regard to pathological findings, the supplementary MRI-criteria supposed by the diagnostic criteria[2, 3] as well as to a panel of supplementary MRI characteristics proposed by the literature and the authors.

The supplementary MR-criteria supposed by the diagnostic criteria from 2013[2] were further specified as published elsewhere[3]:

- Empty sella and hernitation of the suprasellar cisterne were defined according to Yuh et al.[4]. We operationalized empty sella as an enlarged sella with less than 2 mm visible pituitary gland tissue in the sagittal plane and herniation of the suprasellar cisterne as herniation of >1/3 of the sella height. When available, we used pituitary gland sequences.

© 2024 Beier D et al. *JAMA Network Open.*

- We defined distension of the optic nerve sheath as a >2 mm distension of the perioptic subarachnoid space in the coronal or axial plane on T2w or fat suppressed sequences. Tortuosity of the optic nerve was evaluated qualitatively in the axial or sagittal plane on T2w or FLAIR sequences.

- Flattening of the posterior aspect of the globe was qualitatively evaluated in the axial plane on T2w sequences without a predefined cut-off.

- The transverse sinuses were evaluated according to Carvalho et al.[5]. Stenoses were graded on each side as < 33 % stenosis (score: 1), 33-66 % stenosis (score: 2) or > 66 % stenosis (score: 3). Hypoplasia or agenesia (score: 4) was also evaluated, with hypoplasia being defined as the full-length diameter of the transverse sinus being < 1/3 of the superior sagittal sinus. For each patient, the Index of Transverse Sinus Stenosis (ITSS) score was calculated (ITSS = degree of stenosis of the right transverse sinus x degree of stenosis of the left transverse sinus)[5]. A score of ≥ 4 was defined as cut-off for relevant reduction of the bilateral total venous diameter with regard to IIH.

Supplementary MRI characteristics proposed by the literature and the authors:

- The length and width of the ocular globe were evaluated in the axial plane using thin-cut fat suppressed orbita sequences if available; the maximum length and width in the axial plane is given (mm).

- Optic disc protrusion was evaluated qualitatively in the axial plane using thin-cut fat suppressed orbita sequences. As routine MRI is not reliable with regard to this neuroimaging sign, patients were classified as ‘missing data’ and excluded from the analysis, if thin-cut fat suppressed orbita sequences were not available.

- The diameter of the optic nerve canal, the caliber of the optic nerve, the optic nerve sheath and the perineural subarachnoid space were evaluated in the axial plane using thin-cut fat suppressed orbita sequences if available; the maximum value in the axial plane is given (mm).

© 2024 Beier D et al. *JAMA Network Open.*

- Posterior displacement of the pituitary stalk was qualitatively evaluated in the sagittal plane using cerebral MRI or thin-cut pituitary gland sequences if available.

- Arachnoid pits were evaluated qualitatively in the axial plane. Arachnoid pits are arachnoid granulations that penetrated the dura but failed to migrate normally into the venous sinuses.

- Small meningoceles were evaluated qualitatively in the axial plane.

- The inferior position of the cerebellar tonsils was evaluated in the sagittal plane, the maximum is given (mm).

- The Evan's index is defined as the ratio of the maximum width of the frontal horns of the lateral ventricles and the maximum internal diameter of the skull at the same level employed in axial MRI, adjusted for sex and age[6]. The Evan's index can be used to evaluate the presence of slit ventricles.

<u>Statistical analyses used to develop an MRI-score based on our own data predicting papilledema/IIH</u>

To identify crucial MRI signs associated with papilledema, we used multivariate logistic regression using LASSO (least absolute shrinkage and selection operator) regularization for sparse shrinkage of regression coefficients.

Binary outcome was papilledema. The following parameters with significant association in the univariate analyses were used: (1) uni- or bilateral flattening of the posterior aspect of the globe present, (2) uni- or bilateral distension of the perioptic subarachnoid space present, (3) uni- or bilateral optic nerve disc protrusion present and (4) uni- or bilateral transverse sinus stenosis present along with the four categoric variables (5) herniation of the suprasellar cistern present; a total of eight parameters including the intercept. The LASSO was tuned over a grid of regularization parameters chosen by the software by selecting the regularization which minimized the out-of-sample binomial deviance as estimated by 5-fold cross validation.

© 2024 Beier D et al. *JAMA Network Open.*

Model performance was summarized in terms of discrimination as measured by the area under the receiver operating characteristics curve (AUC) along with the E_max statistic proposed by Harrell[7,8]. The latter is calculated by smoothing the relationship between the model predicted risk of positive diagnosis and the observed diagnosis using a locally weighted scatterplot smoothing (LOWESS) yielding a calibration curve. $E_{max}(A, B)$ is then defined as the largest absolute deviation between the predicted and calibrated risk of positive diagnosis for calibrated risks in the range from AA to BB. By E_{max}, i.e. omitting the range, we mean the statistic over the widest range, $E_{max}(0,1)$. We refer to the model performance on the original data set as the apparent performance.

To address the problem of missing predictors, single imputation was performed using chained equations. In chained equations, an imputation model must be specified for each given variable as a function of the remaining variables. We used a logistic regression model to impute binary predictors while herniation of the suprasellar cistern was imputed using predictive mean matching by sampling from the two nearest neighbors. First, a single imputed data set was constructed and the primary analysis repeated to yield estimates of the apparent imputed model performance. Imputations were checked by inspection of tabulated distributions. Imputation was performed and distributions of predictors were compared in observed, imputed, and completed observations. For transverse sinus stenosis, distension of the perioptic subarachnoid space and optic nerve disc protrusion, quite different distributions were seen in imputed compared to observed datasets ('No' much more prevalent). Thus, for transverse sinus stenosis, only 37/191 were imputed and the completed distribution was not much different, and this was similar for distension of the perioptic subarachnoid space. However, imputation changed the optic nerve disc protrusion distribution (120/191 are imputed).

Bootstrapping was performed to assess internal validity based on 200 replications[9]. For each bootstrap sample taken from the original data set, the imputation and primary analysis was repeated using the imputed bootstrap sample as a training set, and the imputed original data set as the test set. The difference in performance between training and test set is an estimate of the optimism, which is stabilized by averaging over bootstrap samples. Finally, optimism-corrected

© 2024 Beier D et al. *JAMA Network Open.*

estimates of imputed model performance are obtained by subtracting the optimism from the apparent performance.

In clinical literature it is sometimes preferred not to include outcomes when performing imputations, despite it being the recommended practice in statistical literature[10, 11]. We did not include the diagnosis outcome in the imputations described above, but as a sensitivity analysis the outcome was included in the imputation model, and the primary analysis was repeated on this imputed data.

When imputations are performed without accounting for the outcome, the association between outcome and predictor is diluted in the imputed observations. We therefore expect the imputations described above to yield conservative estimates of association between diagnosis and explanatory variables, the association diminished proportional to the number of missing data points.

Analyses were performed using Stata 17.0 (SE).

Detailed information on the three independent international cohorts used for external validation:

We chose 3 international cohorts for external validation comprising patients without IIH (cohort 1), patients diagnosed at a tertiary center with different severity of IIH (cohort 2: mild to severe IIH; cohort 3: severely affected patients, referred to the tertiary center for endovascular treatment).

- Cohort 1: 'Outpatients without papilledema': outpatients aged ≥ 18 years undergoing MRI scans for any clinical indication, published elsewhere (n=291, patients without papilledema)[12]. The diagnostic work-up included MRI, non-mydriatic fundus photography of both eyes, questions about the presence of headaches, visual symptoms, and ocular history and their medical records were reviewed for ocular, neurologic, and medical history including previous neurosurgery[12]. Demographic characteristics including age, sex, and BMI were recorded as well. MRI was performed on a 3-T unit (MAGNETOM Skyra, Siemens Healthineers) using a standard head coil and included unenhanced sagittal T1-weighted and axial T2-weighted sequences for all patients[12]. One masked subspecialty-

© 2024 Beier D et al. *JAMA Network Open.*

certified neuroradiologist systematically reviewed all imaging for 10 radiographic signs that were described to be associated with IIH: empty sella (defined as a pituitary grade of ≥3[4], protrusion of the optic nerve head, flattening of the posterior sclera, increased perioptic CSF, vertical tortuosity of the intraorbital optic nerve, enlaged Meckel caves, cephaloceles, cerebellar tonsillar ectopia more than 5mm below the foramen magnum. For patients undergoing contrast-enhanced imaging, the presence of bilateral transverse venous sinus stenosis with at least 50% narrowing and optic nerve head enhancement was also determined[12].

- Cohort 2: 'IIH patients (Austria)': IIH patients aged ≥ 18 years from the Department of Neurology, University Hospital of Vienna, Austria (n=84; Supplementary Table 3) were gathered retrospectively in a database as published elsewhere[13]. The diagnostic work-up comprised detailed medical history with focus on IIH-related symptoms, ophthalmological assessment (visual acuity, fundoscopy, perimetry and optical coherence tomography with optional ocular ultrasonography)[13]. MRI was done using T1 or T2w sequences[13]. All patients received MR-venography. Patients were diagnosed according to the 2013 diagnostic criteria[1]. MRI characteristics were evaluated by a single blinded expert neuroradiologist (W.M.) according to the same definitions as used for our prospective Danish cohort in this manuscript.

- Cohort 3: 'Difficult-to-treat IIH patients (USA)': IIH patients aged ≥ 18 years referred to the Department of Neurology, Thomas Jefferson University, Philadelphia, USA for endovascular treatment due to transverse venous sinus stenosis (n=20; Supplementary Table 4). Patients were diagnosed according to the 2013 diagnostic criteria[1]. MRI characteristics were evaluated according to the same definitions as used for our prospective Danish cohort in this manuscript. Data was retrospectively reviewed from January 2021 to December 2022. Out of the 78 records identified, 27 patients were excluded due to prior brain and spine surgeries. Of the remaining 51 patients, 20 underwent venous manometry and were included in the study. An institutional diagnostic

© 2024 Beier D et al. *JAMA Network Open.*

protocol was developed to retrospectively evaluate the radiographic features. Four neuro-radiologists were blinded and analyzed the radiographic features on available brain (and/or orbit) MRI and brain MR venography. In cases where discrepancies were noted among individual reviews, the radiologists discussed until a consensus was reached. Neuroimaging findings were graded based on available images, and cases meeting 3 out 4 radiographic parameters were considered positive.

© 2024 Beier D et al. *JAMA Network Open.*

eReferences

1. von Elm E, Altman DG, Egger M, et al. The Strengthening the Reporting of Observational Studies in Epidemiology (STROBE) statement: guidelines for reporting observational studies. Lancet 2007;370:1453-1457.

2. Friedman DI, Liu GT, Digre KB. Revised diagnostic criteria for the pseudotumor cerebri syndrome in adults and children. Neurology 2013;81:1159-1165.

3. Korsbaek JJ, Jensen RH, Hogedal L, Molander LD, Hagen SM, Beier D. Diagnosis of idiopathic intracranial hypertension: A proposal for evidence-based diagnostic criteria. Cephalalgia 2023;43:3331024231152795.

4. Yuh WT, Zhu M, Taoka T, et al. MR imaging of pituitary morphology in idiopathic intracranial hypertension. J Magn Reson Imaging 2000;12:808-813.

5. Carvalho GB, Matas SL, Idagawa MH, et al. A new index for the assessment of transverse sinus stenosis for diagnosing idiopathic intracranial hypertension. J Neurointerv Surg 2017;9:173-177.

6. Sari E, Sari S, Akgun V, et al. Measures of ventricles and evans' index: from neonate to adolescent. Pediatr Neurosurg 2015;50:12-17.

7. Harrell Jr. FE, Lee KL, Mark DB. Multivariale Prognostic Models: Issues In Developing Models, Evaluating Assumptions and Adequacy, and Measuring and Reducing Errors. Statistics in Medicine 1996;15:361-387.

8. Austin PC, Steyerberg EW. The Integrated Calibration Index (ICI) and related metrics for quantifying the calibration of logistic regression models. Stat Med 2019;38:4051-4065.

9. Efron B. Estimating the error rate of a prediction rule: improvement on cross-validation. J Journal of the American statistical association 1983;78:316-331.

10. Sterne JA, White IR, Carlin JB, et al. Multiple imputation for missing data in epidemiological and clinical research: potential and pitfalls. BMJ 2009;338:b2393.

11. White IR, Royston P, Wood AM. Multiple imputation using chained equations: Issues and guidance for practice. Stat Med 2011;30:377-399.

© 2024 Beier D et al. *JAMA Network Open.*

12. Chen BS, Meyer BI, Saindane AM, Bruce BB, Newman NJ, Biousse V. Prevalence of Incidentally Detected Signs of Intracranial Hypertension on Magnetic Resonance Imaging and Their Association With Papilledema. JAMA Neurol 2021;78:718-725.

13. Pruckner P, Mitsch C, Macher S, et al. The Vienna idiopathic intracranial hypertension database-An Austrian registry. Wien Klin Wochenschr 2023.

© 2024 Beier D et al. *JAMA Network Open.*
