# Supplementary material for: Magnetic Resonance Imaging Signs of Idiopathic Intracranial Hypertension
Source: JAMA Netw Open. 2024 Jul 3;7(7):e2420138. doi: 10.1001/jamanetworkopen.2024.20138 (PMC11223000; doi:10.1001/jamanetworkopen.2024.20138)
Supplement: Supplement 2. — Data Sharing Statement [file jamanetwopen-e2420138-s002.pdf]

Data Sharing Statement

Beier. Magnetic Resonance Imaging Signs of Idiopathic Intracranial Hypertension. *JAMA Netw Open*. Published July 03, 2024. doi:10.1001/jamanetworkopen.2024.20138

Data
Data available: No

Additional Information
Explanation for why data not available: Data availability Individual, de-identified participant data can be shared with qualified researchers who provide a methodologically sound proposal. It is a legal requirement that a data processing agreement is signed and approved by the data protection office in the Region of Southern Denmark. Raw imaging data are shared locally for technical and legal reasons. Data are available 2 years after publication. Proposals should be directed to the corresponding author.
